# Knee biomechanics variability before and after total knee arthroplasty: an equality of variance prospective study

**DOI:** 10.1038/s41598-024-52965-w

**Published:** 2024-02-01

**Authors:** Erik Kowalski, Danilo S. Catelli, Geoffrey Dervin, Mario Lamontagne

**Affiliations:** 1 Faculty of Health Sciences, University of Ottawa, Ottawa, ON Canada; 2 Department of Movement Sciences, KU Leuven, Leuven, Belgium; 3 Division of Orthopaedic Surgery, The Ottawa Hospital, Ottawa, ON Canada

**Keywords:** Musculoskeletal system, Randomized controlled trials

## Abstract

This study evaluated gait variability in patients before and after total knee arthroplasty (TKA) using the equality of variance method to determine where variability differences occur in the movement cycle. Twenty-eight patients underwent TKA with cruciate-sacrificed implants. Patients underwent motion analysis which measured knee biomechanics as they walked overground at their preferred pace before and 12 months after TKA. Equality of variance results were compared with 14 healthy controls of similar age. Before surgery, patients had reduced knee extension moment variability throughout the early stance phase (4–21% gait cycle, *p* < 0.05) compared to controls. Knee power variability was lower preoperatively compared to controls for most of the stance phase (0–13% and 17–60% gait cycle, *p* < 0.05). Sagittal knee moment and power variability further decreased following TKA. Knee extension moment variability was lower postoperatively throughout stance phase compared to preoperatively (4–22% and 36–60% gait cycle, *p* < 0.05) and compared to controls (4–30% and 45–60% gait cycle, *p* < 0.05). Knee power variability remained lower following TKA throughout stance phase compared to preoperatively (10–24% and 36–58% gait cycle, *p* < 0.05) and controls (3–60% gait cycle, *p* < 0.05). TKA patients may be less stable, and this may be in part due to an unresolved adaptation developed while awaiting TKA surgery and the cruciate sacrificing design of the implants utilized in this study.

## Introduction

Pain and impaired mobility are a daily reality for those living with symptomatic knee osteoarthritis (OA)^1^. As knee OA progresses, greater pain and stiffness occur at the knee joint, which ultimately requires surgical intervention with a total knee arthroplasty (TKA). Despite TKA being a successful surgery, 20% of patients remain dissatisfied after their TKA and often report loss of stability, decreased functional outcomes, reduced knee range of motion, greater difficulty performing daily activities compared to their age-matched peers^2^, and their gait becomes less variable^3^. The question remains as to why patients are still unsatisfied, which could be related to knee instability after TKA.

Orthopaedic surgeons evaluate knee joint instability by assessing knee joint laxity; however, self-reported instability is unrelated to knee joint laxity^4^. Greater knee joint instability can cause a loss of balance and lead to an eventual fall^5^. The measure of knee joint laxity assesses static stability but falls generally occur during movements, implying deficits in dynamic stability^6,7^. Researchers have assessed the dynamic stability of the knee by evaluating the variability of temporospatial and knee biomechanical measures during gait^6,8^.

Gait variability is the amplitude of the fluctuations in the time series with respect to the mean of kinematic (e.g., joint angles) or kinetic (e.g., joint moments) measurements^9^. Several methods to estimate the amount of gait variability are routinely used and include standard deviation^10^, Lyapunov exponent^6^, or the coefficient of variation (CV)^11,12^. Methods such as standard deviation and coefficient of variation provide a measure of global variability, which integrates the variability across the time domain to yield a single scalar value. A review on gait variability in patients with knee OA highlighted that variability remains lower than healthy controls before and after a TKA^3^. However, this was determined using single scalar values of gait variability, so it is unknown where in the gait cycle these variability differences occur. Understanding where significant differences in variance occur in a movement cycle may identify certain motor impairments. This could help influence rehabilitation interventions in individuals with knee OA before or after undergoing a TKA.

Recent studies introduced the equality of variance test, which provides a time-point measurement of data variance inequality for kinetic and kinematic variables^13,14^. Using this method, it can identify where in the movement cycle variability differences occurred. This may be more sensitive and uncover additional variability differences, which typical global variability measurement may miss.

To the researchers’ knowledge, no study has evaluated gait variability in patients before or after TKA using the equality of variance test or examined the effect of implant design on gait variability. Therefore, this study aimed to evaluate overground walking in knee OA patients before and after a TKA to assess the variability of knee joint angles, moments and powers using the equality of variance method, and compared them to a group of healthy participants of similar age. It was hypothesized that differences in knee biomechanics variability would present during single-limb support. During this phase in the gait cycle, all the body’s weight is supported on a single limb so that it requires the greatest amount of stability.

## Results

### Demographics, walking speed and KOOS

No significant differences in age existed between the groups (*p* = 0.617) (Table 1). Body mass index (BMI) was statistically different between the groups (*p* = 0.014). The healthy control (CTRL) group (25.1 ± 2.0) had lower BMI compared to the TKA-Pre (28.8 ± 3.7, *p* = 0.015) and TKA-Post (28.4 ± 4.0, *p* = 0.032). No significant difference in walking speed existed between the groups (*p* = 0.056) (Table 1).

**Table 1:** Group mean (SD) demographic, walking speed, and Knee injury and Osteoarthritis Outcome Score (KOOS) values.

	TKA Pre	TKA Post	CTRL	One way ANOVA outcomes		
				F	Significance	Effect size (η^2^)
Number of participants (n)	28	28	14	–	–	–
Sex (female/male)	12/16	12/16	7/7	–	–	–
Age (years)	63.2 (6.7)	64.0 (6.7)	64.4 (5.6)	0.486	0.617	0.015
Body Mass Index (kg/m^2^)	28.8 (3.7)^§^	28.4 (4.0)^§^	24.9 (2.1)	4.570	**0.014**	0.125
Walking speed (leg-length normalized)	1.33 (0.25)	1.37 (0.22)	1.51 (0.18)	1.281	0.285	0.040
KOOS						
Symptoms	42.5 (15.1)*^§^	75.0 (20.3) *^§^	98.7 (2.6)	63.084	** < 0.001**	0.653
Pain	49.5 (15.7)*^§^	85.9 (11.3)*	98.6 (3.2)	95.447	** < 0.001**	0.740
Function in daily living	59.0 (19.3)*^§^	92.3 (8.7)*	100.0 (0.0)	61.497	** < 0.001**	0.647
Function in sport and recreation	25.5 (20.2)*^§^	65.0 (22.1)*^§^	100.0 (0.0)	76.574	** < 0.001**	0.696
Quality of Life	22.1 (15.1)*^§^	69.2 (18.7)*^§^	100.0 (0.0)	137.923	** < 0.001**	0.805

Significant values are in [bold].

Bonferroni post hoc comparisons represented by: * significant (*p* < 0.05) within-group difference between pre- and post-operative TKA visits, and ^§^ represents significant (*p* < 0.05) difference from CTRL.

All Knee Injury and Osteoarthritis Outcome Score (KOOS) subscale scores significantly (*p* < 0.05) increased from pre-operative to 12-month post-operative visits (Table 1). However, Post-TKA remained with lower symptoms, function in sports and recreation, and quality of life subscales scores compared to the CTRL group (*p* < 0.05).

### Coefficient of variance (CV) analysis for simple scalars

Pre-operatively the TKA group had lower step length variability compared to Post-TKA (− 1.1, 95% CI (− 2.2 to − 0.1), *p* = 0.45), as well as for stride length variability (− 2.8, 95% CI (− 5.1 to − 0.6), *p* = 0.016). Post-operatively the TKA group had greater sagittal knee angle (0.4, 95% CI (0.0 to 0.7), *p* = 0.026) compared to the controls. Knee power variability was significantly greater Pre-TKA (29.0, 95% CI (8.8 to 49.1), *p* = 0.002) and Post-TKA (25.3, 95% CI (5.2 to 45.5), *p* = 0.009) compared to the controls (Table 2).

**Table 2:** Group mean (SD) coefficient of variation values (unitless).

	TKA Pre	TKA Post	CTRL	One way ANOVA outcomes		
				F	Significance	Effect size (η^2^)
Temporospatial						
Speed	3.5 (2.6)	4.2 (2.6)	3.8 (4.0)	0.956	0.390	0.028
Step length	2.2 (1.3)*	3.3 (2.6)*	2.1 (1.6)	0.323	0.725	0.010
Stride length	3.0 (2.0)*	5.8 (5.8)*	3.3 (2.7)	0.074	0.929	0.002
Step width	13.2 (8.5)	12.9 (9.5)	13.6 (8.1)	0.038	0.963	0.001
Stride time	2.5 (1.9)	2.4 (1.5)	2.8 (3.0)	1.648	0.200	0.047
Step time	3.5 (2.7)	3.6 (1.9)	4.4 (5.7)	2.577	0.083	0.071
Knee variables						
Sagittal angle	2.2 (0.5)	2.3 (0.5)^§^	1.9 (0.3)	3.899	**0.025**	0.104
Frontal angle	12.7 (6.9)	14.8 (10.9)	13.6 (7.4)	0.386	0.681	0.011
Sagittal moment	57.2 (28.7)	48.4 (14.1)	30.5 (13.1)	3.120	0.051	0.085
Frontal moment	55.3 (53.9)	54.1 (37.4)	40.7 (24.6)	0.594	0.555	0.017
Power	57.6 (31.9)^§^	54.0 (20.9)^§^	28.6 (14.6)	6.742	**0.002**	0.168

Significant values are in [bold].

Bonferroni post hoc comparisons represented by: * significant (*p* < .05) within-group difference between pre- and post-operative TKA visits, and ^§^ represents significant (*p* < .05) difference from CTRL.

### Equality of variance analysis

**Figure 1:**
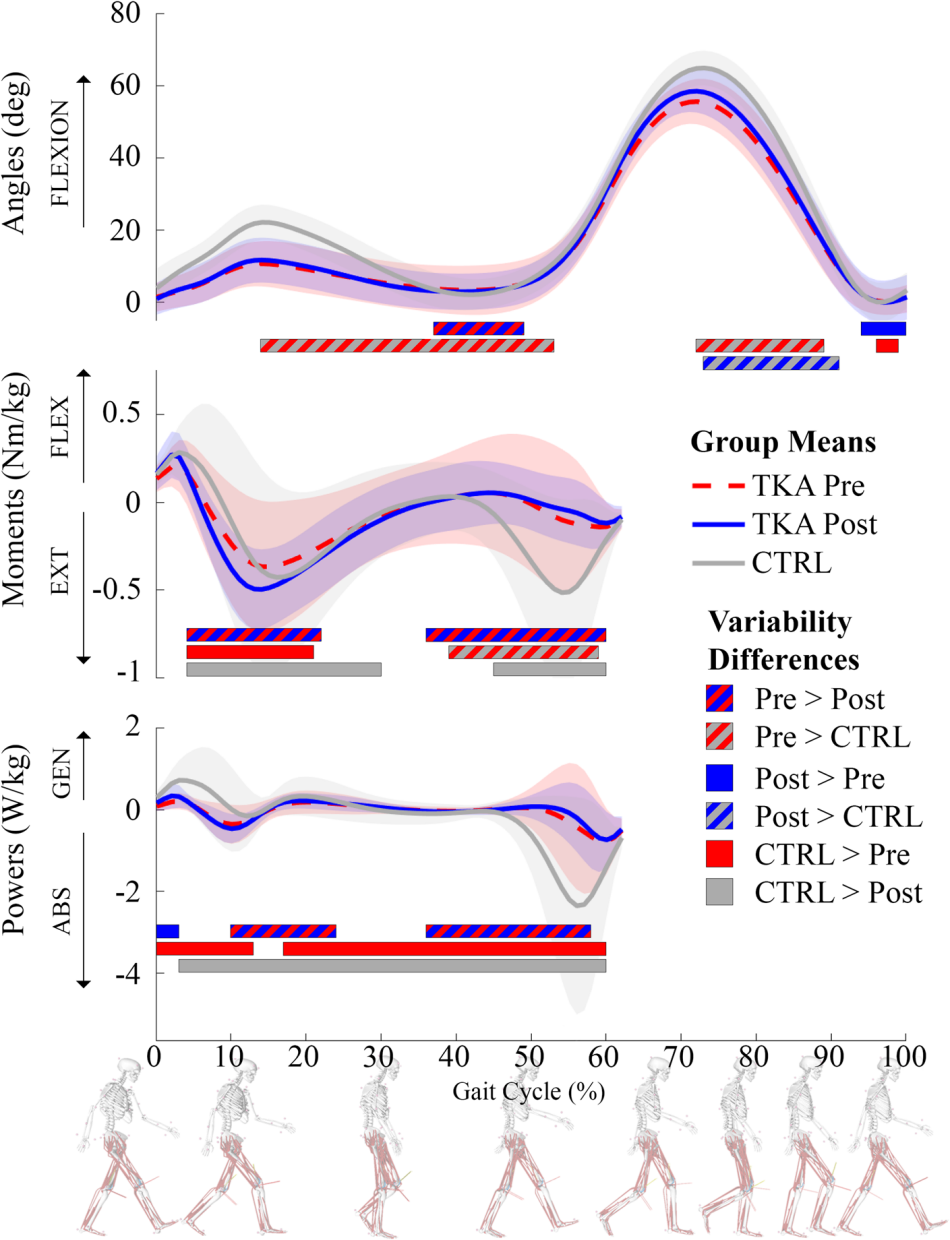
Group means and standard deviations (SD) for sagittal knee angles (degrees), moments (Nm/kg), and powers (W/kg) for the pre-operative TKA, post-operative TKA, and control groups. Horizontal bars represent where in the movement cycle differences in variability occur and identify which group had greater variability (*p* < 0.05).

Knee biomechanics variability between the pre-TKA, post-TKA, and CTRL groups is presented in Fig. 1. Within Fig. 1, the line graph illustrates the group mean knee biomechanics and standard deviation, whereas the horizontal bars represent where in the gait cycle variability was significantly (*p* < 0.05) different between the two groups. In general, patients had less variability post-TKA compared to pre-TKA. Sagittal knee angle variability was lower post-TKA compared to pre-TKA throughout terminal stance (37–49% gait cycle (%)); sagittal knee extension moment variability was lower post-TKA during mid-stance (4–22%) and terminal stance (36–60%); and knee power variability was also lower during mid-stance (10–24%) and terminal stance (36–58%).

Knee biomechanics variability differences between pre-TKA and CTRLs were less consistent. Sagittal knee angle variability was greater in the pre-TKA group throughout most of the single-limb support (14–53%) and at mid-swing (72–89%). Sagittal knee moment variability was greater in the CTRL in the early stance phase (4–21%), but greater in the pre-TKA throughout the terminal stance (39–59%). Knee power variability was greater in the CTRL group throughout loading response (0–13%) and from mid-stance to just before toe-off (17–60%).

Sagittal knee angle variability was greater in the post-TKA group compared to the CTRL group during mid-swing (73–91%). However, the CTRL group had greater sagittal knee moment and knee power variability compared to the post-TKA group. Sagittal knee moment variability was greater throughout mid-stance (4–30%) and during terminal stance prior to toe-off (45–60%). Knee power variability was greater in the CTRL group throughout most of the stance phase (3–60%).

## Discussion

This study aimed to determine gait cycle differences in variability in patients with knee OA before and after undergoing a TKA compared to healthy, similarly aged adults. Whereas previous studies were limited to a simple scalar analysis of variability, which provided an estimate of global variability, this study implemented equality of variance comparison at each interval of the gait cycle^13,14^, which provided the location where the gait cycle differences in variability occurred.

During loading response through early mid-stance, knee OA patients had less sagittal knee moment (4–21%) and less knee power (0–13%) variability compared to healthy individuals (Fig. 1). The healthy controls also had greater variability from mid-stance through to just before toe-off (17–60%). However, the knee OA group did have a period of greater sagittal knee extension moment variability during terminal stance (39–59%) compared to the controls. Following TKA, sagittal knee extension moment variability was lower compared to pre-TKA levels (4–22% and 36–60%) and when compared to the controls (4–30% and 45–60%). Similar reductions in knee power variability following TKA existed when compared to pre-TKA (10–24 and 36–58%) and controls (3–60%). These findings generally align with previous studies showing knee OA patients had lower variability than controls, which decreased after TKA^15–17^.

Evaluating variability can assess system stability. For example, high variability of physiological outcomes, like heart rate variability, is favourable as it reflects greater adaptability and a wider ability to respond. In other situations, high variability is unfavourable as it represents the inability of the physiological control system to regulate a given parameter^18,19^. It has been suggested that during gait, greater kinematic and kinetic variability is favourable as it reflects adaptability^18^. To the best of our knowledge, there is no accepted variability threshold for TKA. In this study, patients walked at a self-selected pace on a flat surface with no obstacles. Pre-TKA patients already had significantly less sagittal knee moment and knee power variability than controls. The further reduction in sagittal knee moment and power variability after TKA, particularly during single-limb support, could mean patients are less stable and less capable of adapting to unanticipated situations while walking.

It is estimated that 7–38% of TKA recipients experience a fall within the first 12 months after TKA^20,21^. Reduced movement adaptability can affect a TKA patient’s ability to respond to walking disturbances, such as stepping over an obstacle or regaining balance after perturbation, which may lead to a fall^22^. However, this variability reduction may result from movement strategies patients utilize to reduce pain and loading on the affected knee. Studies have shown that individuals with OA on the medial compartment of the knee^8^ and after TKA walk with a ‘stiff knee gait pattern’ characterized by prolonged muscle contractions during the stance phase^23^. Patients could increase the co-contraction around the knee to increase stability but reduce their movement variability^17^.

Passive knee stabilization is achieved through knee ligaments, whereas muscles around the knee achieve active stability, and both systems work together to allow reliable knee joint function^24^. These structures provide proprioceptive feedback to allow for the perception of joint movements and the position of joint segments in space. In the knee, proprioception provides three fundamental functions, stabilization during the static posture, protection against excessive and possible injurious movements via reflex responses, and coordination of complex movements and precise knee joint motions^25^. Proprioceptive feedback is negatively affected in individuals with knee OA, but removing damaged structures during TKA has been reported to improve this proprioceptive feedback^3^. However, this improvement in proprioception and joint stability after TKA is accompanied by a further reduction in variability. This may be due to proprioceptive feedback being primarily supplied by the mechanoreceptors in ligaments and muscle tendons^26^. The TKA implants used in this study required the sacrifice of both the ACL and PCL, which could potentially diminish proprioceptive feedback. These changes need to be better understood with predicting joint function and variability.

This study was not designed to determine the source of the decreased movement variability following TKA. However, one possible explanation could be the cruciate sacrificing design of the implants used in this study. The implants in this study (Zimmer Biomet® NexGen® and MicroPort EVOLUTION®) have cruciate sacrificing tibial inserts, meaning the anterior and posterior cruciate ligaments were removed during surgery. These cruciate ligaments play an essential role in the passive stabilization of the knee^24^, so the muscles may have to compensate for this reduced stability by creating more co-contraction, ultimately reducing gait variability^15^. Alternative implant designs preserve the posterior or both cruciate ligaments, improving proprioception more than the cruciate sacrificing TKA^27^. A future study should test this hypothesis by comparing gait variability and muscle activity between various implant designs to determine if the implant design affects gait variability.

Although TKA successfully reduces pain and improves strength around the knee, patients still move with atypical movement patterns suggesting an adapted movement pattern to reduced pain while awaiting TKA^28^. Several strategies to reduce variability have been identified, including stiffening the knee joint through co-contraction, walking slower, or paying more attention while walking^21^, which could have been used by the patients in this study. These strategies do not imply conscious cognitive involvement, as it is suspected that patients do not know how they are adapting or why they are doing so^15,29^. The knee OA patients in this study walked with less variability than the controls (Fig. 1), so the further reduction in variability may have been due to these movement strategies they adopted due to the OA, which were further reduced due to either the implant design or the surgery itself.

This study evaluated TKA implants with cruciate sacrificing inserts, so the findings of this study cannot be generalized to all implant types. It also assessed patients after 12 months of surgery. Patients’ functions may continue to improve beyond this time^30^, so gait variability may increase over time. Variability analysis may be a potential measure of recovery after TKA, so studies should continue to determine if, post-operatively, patients can match their pre-TKA variability levels or even achieve values closer to healthy controls. Future research is necessary to determine the source for the reduction in variability and whether it is due to the cruciate sacrificing design of TKA implants, an unresolved adaptation developed while awaiting TKA, or other unknown reasons.

Like many biomechanical studies, this study had a relatively small sample size which may increase the risk of type II errors^31^. Some evidence has shown that gait variability associated with knee OA is sex-dependent^8^. To overcome this shortcoming, we included the same number of female and male participants in the implant groups and had an equal number of females and males in the control group. The CTRL group had significantly lower BMI than the TKA group at both pre- and post-operative visits. Although this likely did not influence the KOOS findings^32^, it may partially explain differences in variability as a recent study found that stride length CV increased significantly as body fat percentage increased^33^.

In conclusion, this study identified that patients with knee OA had reduced knee moment variability throughout the stance phase compared to healthy participants of similar age. Knee moment and power variability further decreased following TKA and could not provide movement variability like the controls.

## Methods

### Participants

The University of Ottawa Health Sciences and Sciences Research Ethics Board and Ottawa Health Science Network Research Ethics Board approved this randomized control study by the U.S. Food and Drug Administration, and it has been registered in the ClinicalTrials.gov database (NCT02589197, 28/10/2015). To be eligible for participation, patients had to meet the inclusion and exclusion criteria fully. All methods were carried out following relevant guidelines and regulations, and all participants provided written informed consent.

For inclusion, participants were between the ages of 45 and 75 at the time of enrollment and were willing and able to complete required study visits and assessments. Exclusion criteria for all participants included having a BMI and waist circumference measurements > 35 kg/m^2^ and 102 cm respectively for men, and > 35 kg/m^2^ and 88 cm respectively for women^34^; any past or present condition, which in the opinion of the investigators may impact gait; and had a previous joint replacement of the enrolled knee or other lower limb joint replacement. TKA participants could not have a degenerative condition (other than OA in the enrolled knee) impacting joints of the lower extremities. Healthy controls could not have a degenerative condition affecting the lower extremity joints.

**Figure 2:**
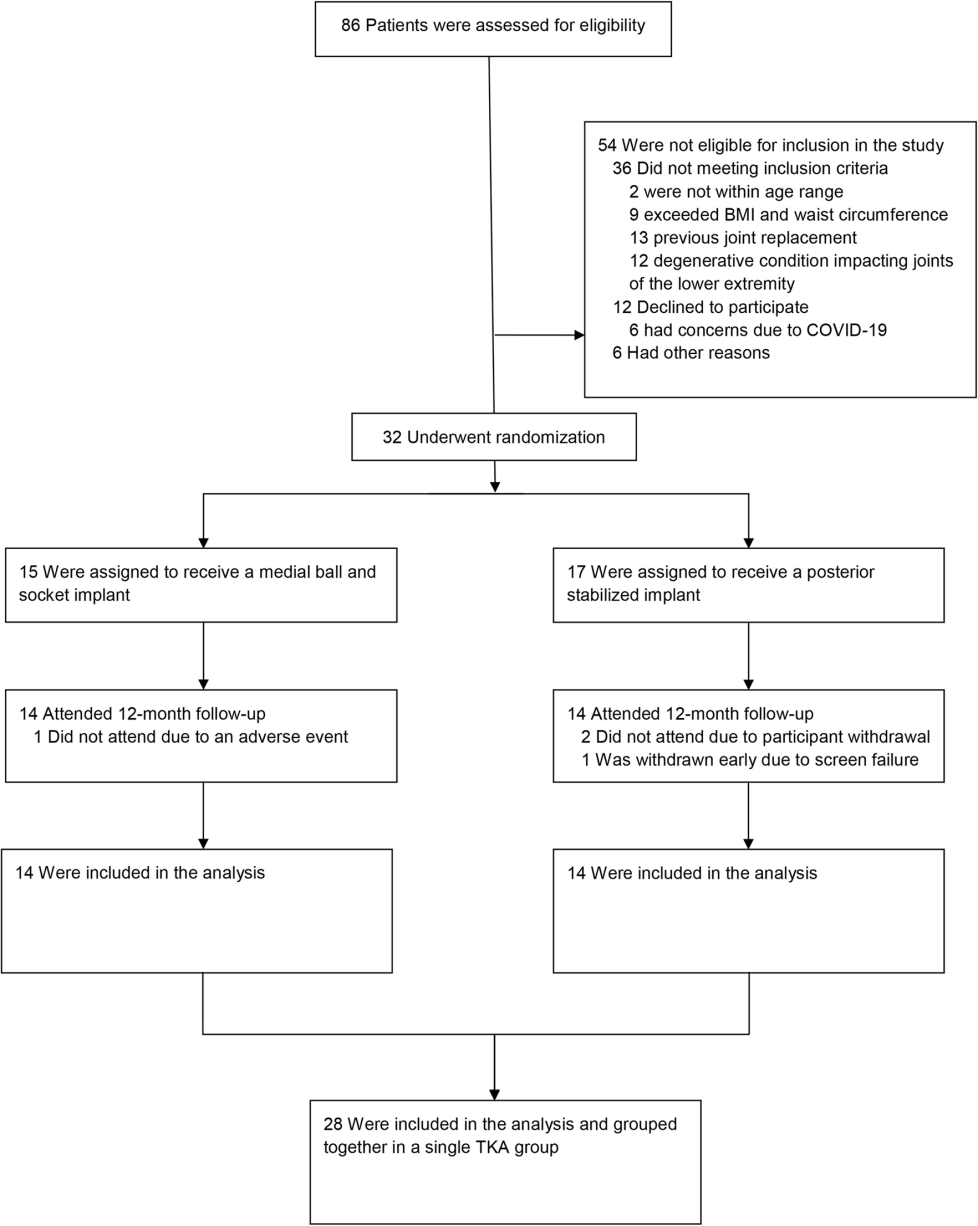
Consolidated Standards of Reporting Trials (CONSORT) flow diagram for enrolled patients.

Eighty-six symptomatic patients with severe knee OA (Kellgren and Lawrence grade 4)^35^ scheduled for TKA were screened. Fifty-four cases were not eligible for participation; their reasons are outlined in Fig. 2. Thirty-two patients fully met the inclusion criteria and were randomly assigned to receive either a MicroPort EVOLUTION® Medial Pivot System with Cruciate Sacrificing tibial inserts or Zimmer Biomet NexGen® TKA System with posterior stabilizer inserts (Fig. 2). Patients were randomized as this study was part of a larger project (clinicaltrials.gov—NCT02589197) which compared the two implant groups. However, the current study evaluated TKA patients compared to controls, and therefore, grouped both implant types into a single group (TKA).

A total of 28 (14 MicroPort EVOLUTION®, 14 Zimmer Biomet NexGen®) patients completed the 12-month follow-up and were included in the final analysis. Fourteen healthy, similarly aged controls were recruited from the community and included in the study (Table 1). TKA and CTRL groups completed the Knee Injury and Osteoarthritis Outcome Score (KOOS) questionnaire at each visit^36^.

### Surgery

A senior arthroplasty surgeon (GFD) performed all surgical procedures, and all TKA patients followed the same protocol. After a suitable anesthetic, patients were given an appropriate weight dose of cefazolin and 15 mg/kg of tranexamic acid intravenously. A midline incision and subvastus approach was performed for all patients^37^. Manual instruments were used with the goal of mechanical neutral alignment with the femur first technique and the goal of the tibial component at a coronal neutral angle. The protocol called for resurfacing all patellae and the PCL was released for all patients. All components were cemented, and tourniquet use was restricted only to the time of cementation and then deflated before closure. Many patients were discharged the same day and continued with eight publicly funded outpatient physical therapy sessions. No patients required additional soft tissue release, and there were no complications or revisions with the surgical cohort.

### Motion analysis

All motion capture was completed using a 10-camera Vicon System (two Vantage V5, eight Vero 2.2, Oxford Metrics, Oxford, UK) sampled at 200 Hz. The capture volume included four force platforms sampled at 1000 Hz: two Bertec force platforms (model FP4060, Bertec Corporation, Columbus, USA) and two Kistler force platforms (model 9286BA, Kistler, Winterthur, Switzerland).

Participants were outfitted with 45 passive-reflective markers placed on anatomical locations using the University of Ottawa Motion Analysis Model (UOMAM)^38^. Participants completed a static trial and five trials of level walking at their preferred, self-selected walking speed. The starting spot for each participant was selected so their first step onto the force platform was always done with the affected limb. The TKA groups completed their first visit within one month of surgery, and 12 months (± 1 month) after surgery, the CTRL group completed one visit.

Motion capture data were processed using Vicon Nexus 2.9.2 software (Oxford Metrics, Oxford, UK). Trajectories were filtered using a Woltring filter with a mean, standard error of 15 mm and force platform data using a 4th-order (zero lag) Butterworth filter with a cut-off frequency of 10 Hz. Gait event detection was done with the assistance of the ground reaction forces. Trials were modelled with the UOMAM^38^, and relevant data were extracted with a custom-written Matlab script (2019b, MathWorks, Natick, USA). Walking speed was extracted and normalized to leg length. Knee variables of interest included sagittal and frontal angles and moments and knee joint power. Knee angles were normalized to 100% gait cycle, whereas knee moments and powers were normalized to 62% stance phase^11^. Data were extracted from the affected limb in the TKA groups and the dominant limb in the CTRL group^19^, defined as the participants’ preferred leg to kick a ball^39^. All five trials were included in the final analyses and were not averaged together.

### Statistical analyses

Scalar variance comparison was completed using the coefficient of variation (CV), which was calculated and compared to the equality of variance results (Eq. 1)^11^.

1 $$CV = \frac{{\sqrt {\frac{1}{N}\mathop \sum \nolimits_{i = 1}^{N} \sigma_{i}^{2} } }}{{\frac{1}{N}\mathop \sum \nolimits_{i = 1}^{N} \left| X \right|_{i} }} \times 100$$

*N* is the number of intervals. *X* is the amplitude of the variable of interest at the *i*th interval. $\sigma_{i}^{2}$ is the standard deviation of *X* at the *i*th interval.

Statistical analyses for the KOOS, CV, and walking speed variables were processed using the SPSS v.27 software (IBM Corporation, Armonk, USA). A One-Way Analysis of Variance with a Bonferroni post-hoc correction was used for the between-group comparisons, and significance was set to *p* < 0.05 for all comparisons. Effect size is reported as ω^2^ for ANOVA.

Knee joint angle, moment, and power variability were compared between the pre-TKA, post-TKA, and control groups using the equality of variance test. Equality of variance was compared between the groups using the ‘gwv1d’ function^13^ in Matlab.

## Data Availability

The data that support the findings of this study are available from MicroPort Orthopedics but restrictions apply to the availability of these data, which were used under license for the current study, and so are not publicly available. Data are however available from the authors upon reasonable request and with permission of MicroPort Orthopedics.

## References

[1] Guccione AA (1994). The effects of specific medical conditions on the functional limitations of elders in the Framingham Study. Am. J. Public Health.

[2] Varadarajan KM (2015). Cruciate retaining implant with biomimetic articular surface to reproduce activity dependent kinematics of the normal knee. J. Arthroplasty.

[3] Smith JW, Christensen JC, Marcus RL, LaStayo PC (2014). Muscle force and movement variability before and after total knee arthroplasty: A review. World J. Orthop..

[4] Schmitt LC, Fitzgerald GK, Reisman AS, Rudolph KS (2008). Instability, laxity, and physical function in patients with medial knee osteoarthritis. Phys. Ther..

[5] Nevitt MC (2016). Symptoms of knee instability as risk factors for recurrent falls. Arthritis Care Res..

[6] Yakhdani HR (2010). Stability and variability of knee kinematics during gait in knee osteoarthritis before and after replacement surgery. Clin. Biomech. (Bristol, Avon).

[7] Reeves NP, Narendra KS, Cholewicki J (2007). Spine stability: The six blind men and the elephant. Clin. Biomech. (Bristol, Avon).

[8] Lewek MD, Scholz J, Rudolph KS, Snyder-Mackler L (2006). Stride-to-stride variability of knee motion in patients with knee osteoarthritis. Gait Post..

[9] Chau T, Young S, Redekop S (2005). Managing variability in the summary and comparison of gait data. J. NeuroEng. Rehabil..

[10] Owings TM, Grabiner MD (2004). Step width variability, but not step length variability or step time variability, discriminates gait of healthy young and older adults during treadmill locomotion. J. Biomech..

[11] Winter DA (1984). Kinematic and kinetic patterns in human gait: Variability and compensating effects. Hum. Mov. Sci..

[12] Ferreira, V., Machado, L. & Roriz, P. *Advances and Current Trends in Biomechanics* (edsBelinha, J. *et al*.) 34–38 (Taylor & Francis Group, 2021).

[13] Kowalski E, Catelli DS, Lamontagne M (2021). A waveform test for variance inequality, with a comparison of ground reaction force during walking in younger vs. older adults. J. Biomech..

[14] Kowalski E, Catelli DS, Lamontagne M (2022). Gait variability between younger and older adults: An equality of variance analysis. Gait Post..

[15] Fallah-Yakhdani HR (2010). Stability and variability of knee kinematics during gait in knee osteoarthritis before and after replacement surgery. Clin. Biomech..

[16] Kiss RM, Bejek Z, Szendrői M (2012). Variability of gait parameters in patients with total knee arthroplasty. Knee Surg. Sports Traumatol. Arthrosc..

[17] Fallah-Yakhdani HR (2012). Determinants of co-contraction during walking before and after arthroplasty for knee osteoarthritis. Clin. Biomech..

[18] Hausdorff JM (2007). Gait dynamics, fractals and falls: Finding meaning in the stride-to-stride fluctuations of human walking. Hum. Mov. Sci..

[19] Hausdorff JM, Rios DA, Edelberg HK (2001). Gait variability and fall risk in community-living older adults: A 1-year prospective study. Arch. Phys. Med. Rehabil..

[20] Chan ACM, Jehu DA, Pang MYC (2018). Falls after total knee arthroplasty: Frequency, circumstances, and associated factors—a prospective cohort study. Phys. Therapy.

[21] Liu Y (2020). A systematic review and meta-analysis of fall incidence and risk factors in elderly patients after total joint arthroplasty. Medicine.

[22] Smith A (1993). Variability in human locomotion: Are repeat trials necessary?. Austr. J. Physiother..

[23] Benedetti MG (2003). Muscle activation pattern and gait biomechanics after total knee replacement. Clin. Biomech..

[24] Abulhasan J, Grey M (2017). Anatomy and physiology of knee stability. J. Funct. Morphol. Kinesiol..

[25] di-Laura-Frattura G (2019). Total knee arthroplasty in patients with knee osteoarthritis: Effects on proprioception. A systematic review and best evidence synthesis. J. Arthroplasty.

[26] Salamanna F (2023). Proprioception and mechanoreceptors in osteoarthritis: A systematic literature review. J. Clin. Med..

[27] Isaac SM (2007). Does arthroplasty type influence knee joint proprioception? A longitudinal prospective study comparing total and unicompartmental arthroplasty. The Knee.

[28] Farquhar SJ, Reisman DS, Snyder-Mackler L (2008). Persistence of altered movement patterns during a sit-to-stand task 1 year following unilateral total knee arthroplasty. Phys. Ther..

[29] Dijksterhuis A, Aarts H (2010). Goals, attention, and (Un)consciousness. Annu. Rev. Psychol..

[30] Cheng K, Dashti H, McLeod G (2007). Does flexion contracture continue to improve up to five years after total knee arthroplasty?. J. Orthopaed. Surg. (Hong Kong).

[31] Knudson D (2017). Confidence crisis of results in biomechanics research. Sports Biomech..

[32] Overgaard A, Lidgren L, Sundberg M, Robertsson O, Annette WD (2019). Patient-reported 1-year outcome not affected by body mass index in 3,327 total knee arthroplasty patients. Acta Orthopaed..

[33] Lee Y, Shin S (2022). The effect of body composition on gait variability varies with age: Interaction by hierarchical moderated regression analysis. Int. J. Env. Res. Public Health.

[34] Ross R (2020). Waist circumference as a vital sign in clinical practice: A Consensus Statement from the IAS and ICCR Working Group on Visceral Obesity. Nat. Rev. Endocrinol..

[35] Kellgren JH, Lawrence JS (1957). Radiological assessment of osteo-arthrosis. Ann. Rheum. Dis..

[36] Roos EM, Lohmander LS (2003). The Knee injury and Osteoarthritis Outcome Score (KOOS): From joint injury to osteoarthritis. Health Qual. Life Outcomes.

[37] Hofmann AA, Plaster RL, Murdock LE (1991). Subvastus (Southern) approach for primary total knee arthroplasty. Clin. Orthopaed. Relat. Res..

[38] Mantovani G, Lamontagne M (2017). How different marker sets affect joint angles in inverse kinematics framework. J. Biomech. Eng..

[39] Chapman JP, Chapman LJ, Allen JJ (1987). The measurement of foot preference. Neuropsychologia.

